# Dynamic Zero Current Method to Reduce Measurement Error in Low Value Resistive Sensor Array for Wearable Electronics

**DOI:** 10.3390/s23031406

**Published:** 2023-01-26

**Authors:** Huanqian Zhang, Jee Chin Teoh, Jianfeng Wu, Longteng Yu, Chwee Teck Lim

**Affiliations:** 1 BME, National University of Singapore, Singapore 119077, Singapore; 2 Shanghai Institute of Microsystem and Information Technology, Chinese Academy of Sciences, Shanghai 200050, China; 3 China Nanhu Academy of Electronics and Information Technology, Jiaxing 314001, China; 4 State Key Laboratory of Bioelectronics, Southeast University, Nanjing 210096, China; 5 Research Center for Humanoid Sensing, Zhejiang Lab, Hangzhou 311121, China

**Keywords:** dynamical zero current, input offset voltage, low value resistive sensor array, measurement error, parasitic resistance, zero potential method

## Abstract

One advantage of a resistive sensor array (RSA) with shared rows (*M*) and shared columns (*N*) is the reduced number of wires from *M × N + 1* to *M + N* which can greatly lessen the complexity and burden on wearable electronic systems. However, the drawback is the crosstalk current effect between adjacent elements, which will lead to high measurement error. Although several solutions have been reported, they mainly focus on RSAs with high resistance (≥100 Ω). There is a lack of research that addresses RSAs with resistor values below 100 Ω. Here, we introduce a new circuit design named the dynamic zero current method (DZCM) to further decrease the measurement error. From the low value RSA test with ideal resistors, the DZCM exhibits lower error than the zero potential method (ZPM). In the case of the error variation ratio of amplifier offset voltage, the DZCM has a 4%/mV (row) to 7%/mV (column) ratio, while the ZPM has an almost 25%/mV (row) to 45%/mV (column) ratio and it increases with array size.

## 1. Introduction

Eutectic gallium indium (EGaIn), an alloy consisting of 75% gallium and 25% indium, is a low viscosity liquid metal at room temperature and has good electrical conductivity [1]. These unique characteristics of EGaIn make it an ideal active component to be embedded in soft sensors. Upon deformation of the sensor microstructure, which is pre-filled with EGaIn, the strain and pressure [2,3,4] exerted can be deduced easily from the measured varying capacitance and resistance.

By encapsulating EGaIn liquid metal with soft elastomers, our group has previously designed and fabricated EGaIn based microfluidic pressure sensors [5,6,7] with relatively low baseline resistance of 10 Ω (no load) which can go up to 200 Ω depending on the loading applied to it. Several examples employing such soft sensors have been demonstrated for healthcare and wearable electronics applications. Many of the current health sensing studies focus on the self-monitoring of personal health data. One such example is the tracking of plantar pressure of individuals with diabetes with diabetic foot ulcers via a flexible pressure sensitive insole embedded with soft sensors.

These sensors are stretchable, conformable and sensitive to mechanical loading and have great potential to be used as building blocks of electronic skins. Undoubtedly, a single sensor is not sufficient to replicate the function of human skin which has closely packed mechanoreceptors with very small receptive fields. To achieve high spatial resolution, a large number of small individual sensors are needed. These sensors are required to be merged firmly to form a compact resistive sensor array (RSA). An RSA enables a high density sensing element network to be configured with minimal wire linkage and thereby confers the least possible burden to the electrical system. One major concern of this RSA architecture is the crosstalk current effect resulting from adjacent unmeasured sensors that give rise to additional measurement error.

**Figure 1:**
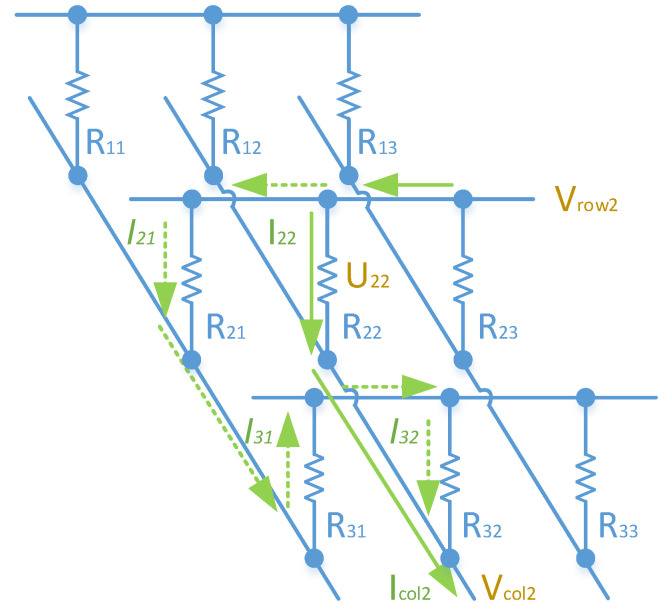
Crosstalk currents induced in the resistor array.

Figure 1 shows a 3 × 3 RSA. To calculate the resistance of the target resistor ‘R_22_′, we need to assess the current flowing through it and the voltage difference across the element, denoted as ‘I_22_′ and ‘U_22_′ accordingly. ‘U_22_′ also equals the voltage difference between the row and column wire ‘V_row2_–V_col2_′. On the other hand, ‘I_22_′ cannot be measured directly. ‘I_col2_′ in the column wire is not equivalent to ‘I_22_′ as ‘I_col2_′ includes crosstalk currents from the adjacent unmeasured resistors. The dashed arrow lines in Figure 1 illustrate one example of these crosstalk currents. Consequently, the actual value of ‘R_22_′ cannot be calculated easily from ‘I_col2_′ and ‘U_22_′.

**Figure 2:**
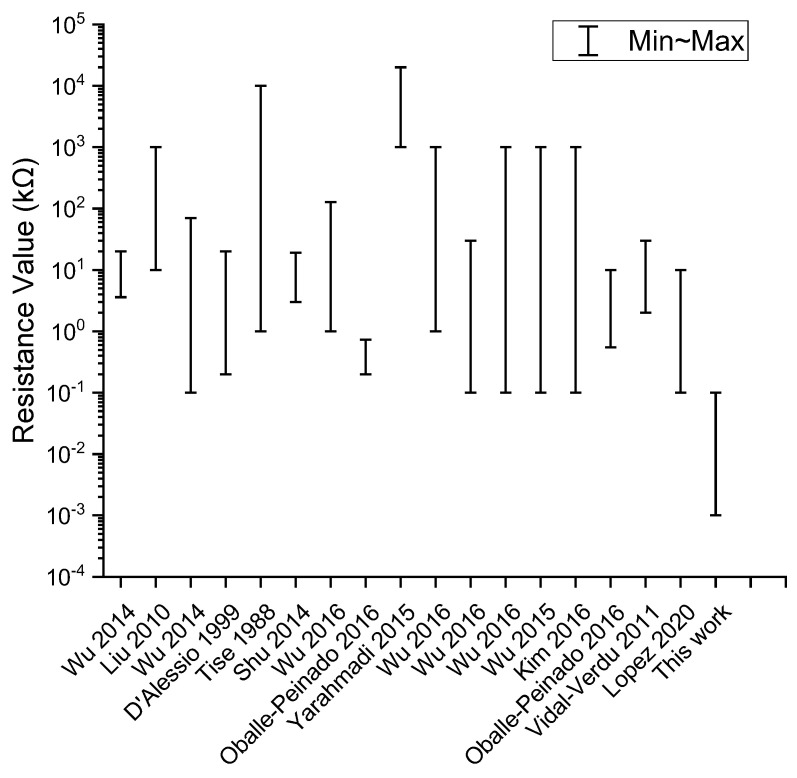
The range of resistance values addressed in different publications [14,15,16,17,19,21,24,25,27,28,29,30,33,34,36].

A number of RSA readout systems have been proposed to eliminate the crosstalk current effect. These approaches include the inserting diode method (IDM) [8,9], inserting transistor method (ITM) [10,11,12,13], passive integrator method (PIM) [14,15,16], resistance matrix approach (RMA) [17], improved RMA [18,19], incidence matrix approach (IMA) [20], voltage feedback method (VFM) [21,22,23,24,25,26,27,28,29,30] and zero potential method (ZPM) [29,31,32,33,34,35,36,37,38,39,40]. However, these systems only cater for RSA designs with high resistance. Electronic networks with low resistance below 100 Ω are often left unaddressed as shown in Figure 2.

The lack in research progress on low resistance networks is partially attributed to the large crosstalk current effect and parasitic effect in the printed circuit board (PCB). In addition, the majority of the mechanical sensors and actuators in the market use high resistance transducers, due to the construction materials, fabrication techniques and sensing mechanisms involved.

Several methods, including the ZPM, VFM and IDM, have been developed to alleviate the crosstalk current effect. Based on a detailed comparative analysis [24], Liu concluded that the ZPM has the best performance, compared to the VFM and IDM. A detailed description of the ZPM is in Appendix A.

Following this introductory section, Section 2 describes a new dynamic zero current method (DZCM) to minimize the measurement error of low value RSAs. Section 3 presents the experiments and discussion. Section 4 provides conclusions.

## 2. Dynamical Zero Current Method

In the discussion in Appendix A, we point out that the ZPM suffers from parasitic effects. The crosstalk current effect is deteriorated due to two reasons:

1. Parasitic resistance from connection wires and PCB wires contributes a large crosstalk current effect.
2. Offset voltage of row and column driving amplifiers induces a crosstalk current effect.

These two issues are difficult to eradicate, as they are caused by the intrinsic feature of these electrical components.

To fix the abovementioned problems, we proposed the DZCM. The DZCM is originated from the ZPM which drives both ends of the adjacent unmeasured resistors to zero potential. Thus, almost no current flows through these adjacent unmeasured resistors. Then, the crosstalk current path will be cut off. In the ZPM, because of the row/column parasitic resistance and row/column amplifier offset voltage, the zero current is not really zero. This amount of current is negligible in a large value RSA, but not so in the case of a low value RSA. This non-zero current must be minimized to reduce the measurement error in the low value RSAs. Based on the fundamental circuit topology of the ZPM, the proposed DZCM includes a feedback network to automatically enforce zero current through each row of the adjacent unmeasured resistors. This feedback feature is also able to flexibly adjust the node potential of the array resistors to match the varying row/column parasitic resistance and amplifier offset voltage in the readout system.

**Figure 3:**
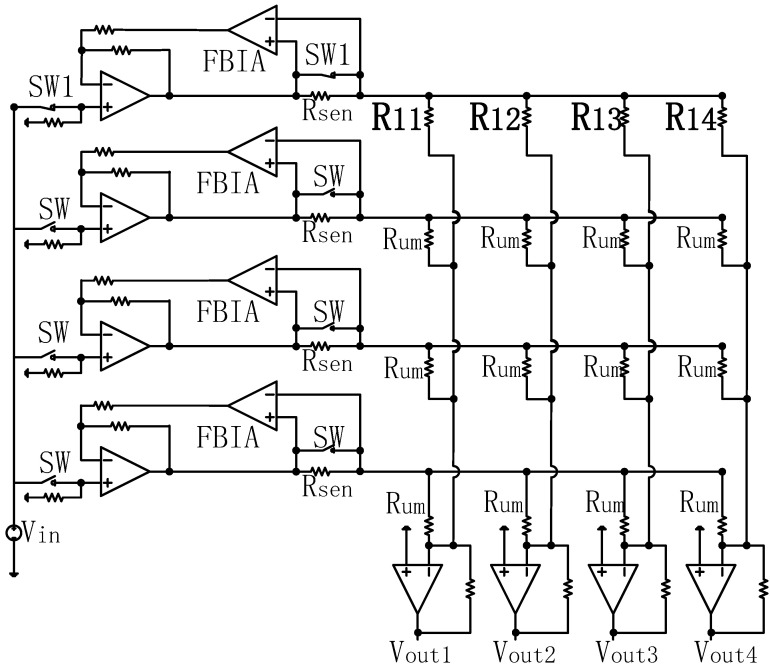
The circuit design of DZCM for a 4 × 4 array.

The DZCM circuit design for a 4 × 4 array is shown in Figure 3. $R_{sen}$ is the current sensing resistor, which converts current to voltage. A feedback instrument amplifier (FBIA) magnifies the voltage across $R_{sen}$. SW1 are the switches with a closed (ON) state for rows containing measuring resistors and SW are the switches with an open (OFF) state for the adjacent unmeasured rows. All amplifiers in Figure 3 are non-ideal.

**Figure 4:**
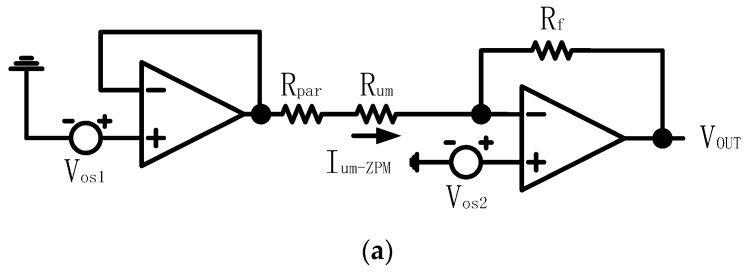

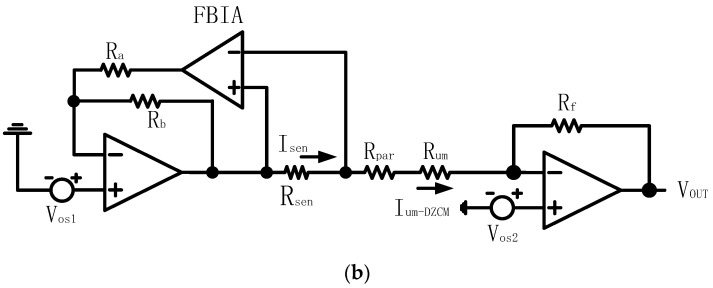
Simplification network with one unmeasured resistor of (**a**) ZPM and (**b**) DZCM.

To explain the characteristics of the ZPM and DZCM more clearly, we simplify the network to just one unmeasured resistor $R_{um}$ to demonstrate the parasitic effect of this single resistor. Figure 4 shows the circuit of this simplified models, all amplifiers in Figure 4 are ideal.

We can derive the current of $R_{um}$ as:

(1a) $$I_{um-ZPM}=\left(V_{os1}-V_{os2}\right)/\left(R_{um}+R_{par}\right)$$

(1b) $$I_{um-DZCM}=I_{sen}=\frac{V_{os1}-V_{os2}}{R_{sen}\left(1+A_{f}\right)+R_{um}+R_{par}}$$

$I_{um-ZPM}$ and $I_{um-DZCM}$ are the currents of the unmeasured resistor for the ZPM and DZCM, respectively. They are expressed in Equations (1a) and (1b) accordingly. $V_{os1}$ and $V_{os2}$ are the offset voltages of row and column driving amplifiers, $R_{par}$ is the parasitic resistance of row and column wires, $R_{sen}$ is the resistance of the current sensing resistor, $A_{f}$ is the gain of FBIA r and $I_{sen}$ is the current of sensing resistor.

From Equation (1a) for the ZPM and Equation (1a,b) for the DZCM, we can clearly see that $I_{um-DZCM}$ computed from Equation (1b) is smaller than $I_{um-ZPM}$ in Equation (1a) due to the presence of the $R_{sen}\left(1+A_{f}\right)$ term in the denominator.

We assume $A_{f}=1000$, $R_{sen}=1.0\;\Omega$, $R_{um}=10.0\;\Omega$, $R_{par}=1.0\;\Omega$, $R_{a}=R_{b}=1.0\;k\Omega$ $V_{os1}=1.00\;\mathrm{mV}$ and $V_{os2}=-1.00\;\mathrm{mV}$. We can then easily evaluate $I_{um-ZPM}$ and $I_{um-DZCM}$ from Equation (1). $I_{um-ZPM}=0.18\;\mathrm{mA}$ is based on Equation (1a) for the ZPM and $I_{um-DZCM}=0.0019\;\mathrm{mA}$ is based on Equation (1b) for the DZCM. Resulting from $V_{os1}$, $V_{os2}$ and $R_{par}$, the negative feedback in the DZCM decreases its parasitic effect current down to 1% of the parasitic effect current in the ZPM circuit. To put it simply, the DZCM can greatly minimize the measurement error in the low value RSA.

**Figure 5:**
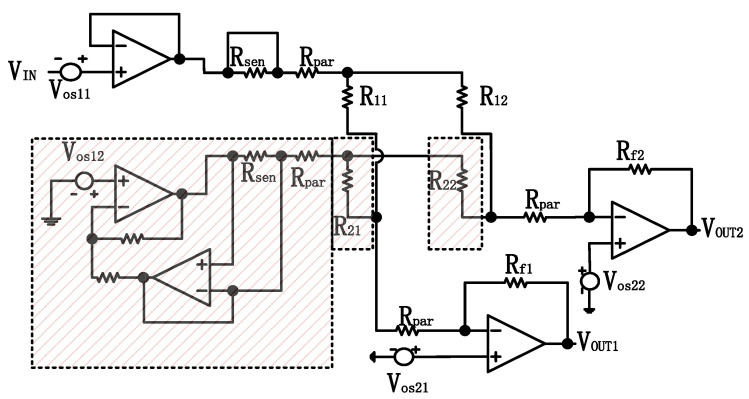
A 2 × 2 array circuit model including parasitic effects and crosstalk current effect for DZCM.

**Figure 6:**
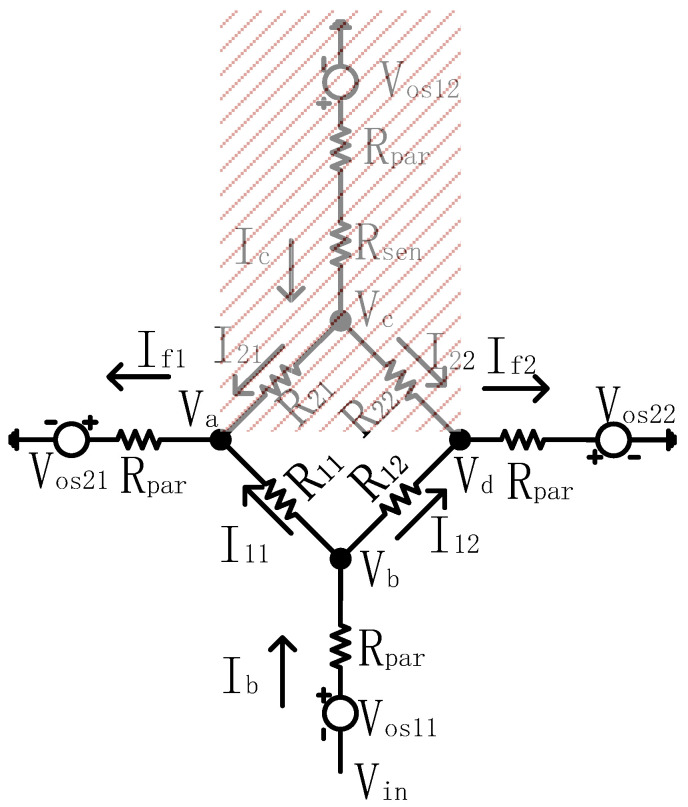
DZCM array network extracted and analyzed with Kirchhoff’s law.

**Figure A3:**
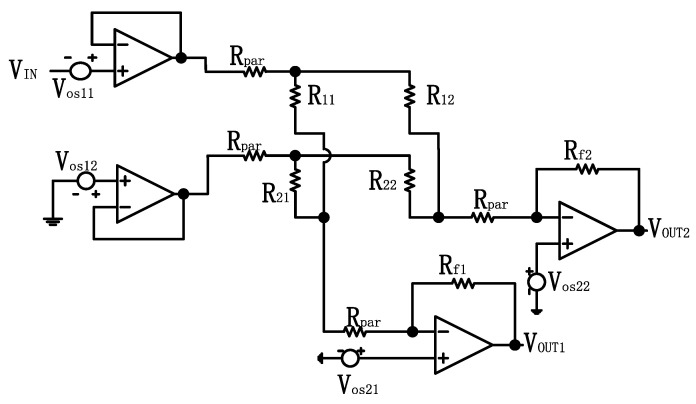
A 2 × 2 array circuit model including parasitic and crosstalk current effects for ZPM.

We further simplify the 4 × 4 array to a 2 × 2 array to quantify the crosstalk current effect in the DZCM, as shown in Figure 5. The reduced array network also includes a parasitic effect originated from $V_{os1}$, $V_{os2}$ and $R_{par}$. Based on the above discussion and Equation (1a) and Equation (1b), the currents of the adjacent unmeasured resistors $R_{21}$ and $R_{22}$ in the DZCM are only 1% of those in the ZPM. Thus, in the DZCM, the crosstalk current effect from $R_{21}$ and $R_{22}$ is minimal and negligible. Compared with Figure A3, $R_{21}$ and $R_{22}$ are shaded in Figure 5 and can be ignored in the Equation (2) formula derivation. The circuit diagram of Kirchhoff’s law, as shown in Figure 6, is used to analyze the DZCM network from Figure 5.

(2a) $$V_{a}=V_{os21}+I_{f1}R_{par}=V_{os21}+I_{11}R_{par}$$

(2b) $$V_{b}=V_{os11}+V_{in}-I_{b}R_{par}=V_{os11}+V_{in}-\left(I_{11}+I_{12}\right)R_{par}$$

(2c) $$V_{d}=I_{f2}R_{par}+V_{os22}=I_{12}R_{par}+V_{os22}$$

(2d) $$V_{b}-V_{a}=I_{11}R_{11}$$

(2e) $$V_{b}-V_{d}=I_{12}R_{12}$$

In order to simplify calculation, we hypothesize that $R_{11}=R_{x}$, $R_{12}=R_{um}$ and $10\cdot R_{par}<R_{um}\approx R_{x}$. After substituting (2a, 2b, 2c) with (2d, 2e), we obtain Equation (3).

(3a) $$I_{11}\left(R_{x}+2R_{par}\right)+I_{12}R_{par}=V_{os11}+V_{in}-V_{os21}$$

(3b) $$I_{11}R_{par}+I_{12}\left(R_{um}+2R_{par}\right)=V_{os11}+V_{in}-V_{os22}$$

Equation (3) is a non-homogeneous linear equation R·I=V and after several solving steps in Appendix B, we have:

(4) $$I_{f1}=I_{11}=-\frac{\left(V_{i1}-V_{os22}\right)R_{par}+\left(V_{os21}-V_{i1}\right)\left(R_{um}+2R_{par}\right)}{\left(R_{x}+2R_{par}\right)\left(R_{um}+2R_{par}\right)-R_{par}{}^{2}}$$

As $R_{par}\ll R_{um}\approx R_{x}$, we can simplify Equation (4) as:

(5) $$I_{f1}=\frac{V_{os11}+V_{in}}{R_{x}}-\frac{V_{os21}}{R_{x}}$$

If we assume $R_{par}\ll R_{um}\approx R_{x}$, Equation (A8) can be written as Equation (6):

(6) $$I_{f1}=\frac{V_{in}+V_{os11}}{R_{x}}-\frac{V_{os12}\left(2R_{um}+R_{x}\right)}{R_{um}R_{x}}-\frac{V_{os21}\left(R_{um}+R_{x}\right)}{R_{um}R_{x}}$$

It is clear to see the Equation (5) is similar to Equation (6), but Equation (5) does not have the term $\frac{V_{os12}\left(2R_{um}+R_{x}\right)}{R_{um}R_{x}}$, which exists in Equation (6). This makes Equation (5) have a lower error resulting from $V_{os12}$.

## 3. Experiments for DZCM/ZPM and Discussion

Various experiments have been designed to evaluate the performances of the ZPM and DZCM under optimum circumstances. The experimental setup is shown in Table 1.

The measurement result from a multimeter of an ideal single resistor is represented as $R_{id}$. Meanwhile, the output amplifier’s voltage of array resistors is measured as $V_{out}$ by a multimeter. Using Equation (A1), we can obtain the resistance value of the resistor of interest, $R_{x}$. The measurement percentage error between them is evaluated as follows:

(7) $$e\%=\frac{R_{id}-R_{x}}{R_{id}}\times 100$$

Experiment **(EXP) A** analyzes the effect of $R_{x}$ on e% in arrays of various sizes when the unmeasured array resistors $R_{um}$ are fixed at their lower and upper limits (i.e., 1 Ω and 200 Ω, respectively) and $R_{par}$ and $v_{os}$ are set to zero.

**EXP B** analyzes the effect of unmeasured array resistors $R_{um}$ on e% in the simplest 2 × 2 array, as $R_{x}$ increases and $R_{par}$ and $v_{os}$ are zero.

**EXP C** analyzes the effect of parasitic resistance of column and row ($R_{parCol}$ and $R_{parRow}$) on e% when the unmeasured array resistors $R_{um}$ and $R_{x}$ are fixed at 1Ω ($V_{in}=10\;\mathrm{mV}$), 50 Ω ($V_{in}=100\;\mathrm{mV}$), 200 Ω ($V_{in}=1000\;\mathrm{mV}$) and offset voltage $v_{os}$ is zero. The reason for increasing $V_{in}$ with increasing $R_{um}$ and $R_{x}$ is to avoid the crosstalk current effect, which will surpass the signal current of $R_{x}$ if $V_{in}$ is fixed to 10 mV and $R_{x}$ increases to 50 Ω or 200 Ω. The array size is set to 6 × 6 and 12 × 12.

**EXP D** analyzes the effect of $v_{os}$ on e% when the unmeasured array resistors $R_{um}$ and $R_{x}$ are fixed at 1 Ω ($V_{in}=10\;\mathrm{mV}$), 50 Ω ($V_{in}=100\;\mathrm{mV}$), 200 Ω ($V_{in}=1000\;\mathrm{mV}$) and $R_{par}$ is set to zero. The array size is set to 6 × 6 and 12 × 12.

### 3.1. Experimental Result for DZCM with Ideal Resistors

**Figure 7:**
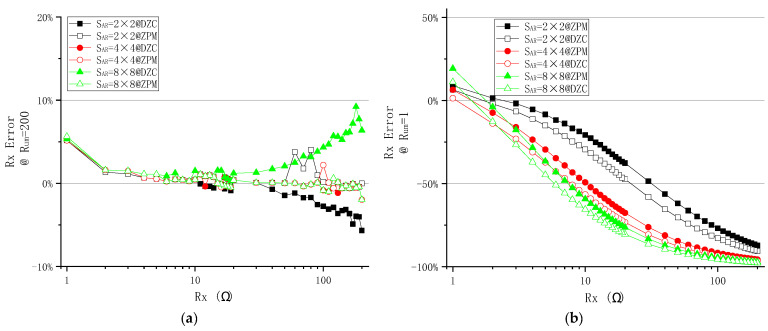
The effect of $R_{x}$ on measurement error of different $S_{AR}$ when (**a**) $R_{um}=200\;Ω$ and (**b**) $R_{um}=1\;Ω$ in DZCM and ZPM (EXP A).

**EXP A**:

As shown in Figure 7a, with $R_{um}=200\;Ω$,

i. the measurement errors of the DZCM and ZPM are found to be comparable when $R_{x}$ falls within the range of 1 Ω to 10 Ω. There is no significant improvement on the system performance by the additional feedback network of the DZCM in this $R_{x}$ range.
ii. As $R_{x}$ goes beyond 10 Ω to 200 Ω, the *e%* of the DZCM is noticeably larger than that of the ZPM in all array sizes due to the underlying crosstalk current effect. The crosstalk current in the DZCM feedback network amounts to 25 µA, while the offset voltage of the feedback amplifier ‘AD623′ is 25 µV and the resistance value of the sensing resistor is 1 Ω. This gives rise to an undesired crosstalk current in $R_{x}$, as, if $V_{in}=10\;\mathrm{mV}$ and $R_{x}=200\;Ω$, we will have $I_{Rx}=10\;\mathrm{mV}/200\;Ω=50\;\mu A$. Meanwhile, the offset voltage of the ZPM is intentionally and manually compensated to zero to minimize the crosstalk current down to zero. We used an adjustable resistor to form a reference voltage divider and connect it to the positive node of row/column amplifiers, shown as $V_{os1}$ and $V_{os2}$, as displayed in Figure 4a. The result shows that the ZPM outperforms the DZCM within this range of $R_{x}$, i.e., 10 Ω to 200 Ω, with notably smaller measurement error. Nonetheless, this crosstalk current effect in the DZCM can be further suppressed by increasing $V_{in}$ or $R_{sen}$ in the feedback network.

**Figure 8:**
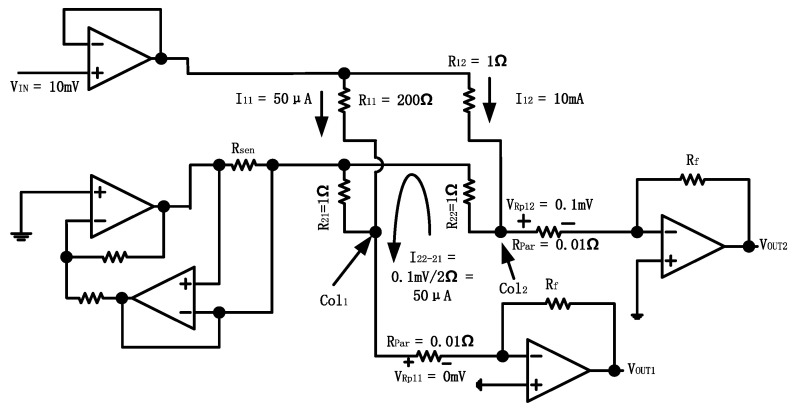
Simplified 2 × 2 array circuit example to demonstrate singular values effect in DZCM.

**Figure 9:**
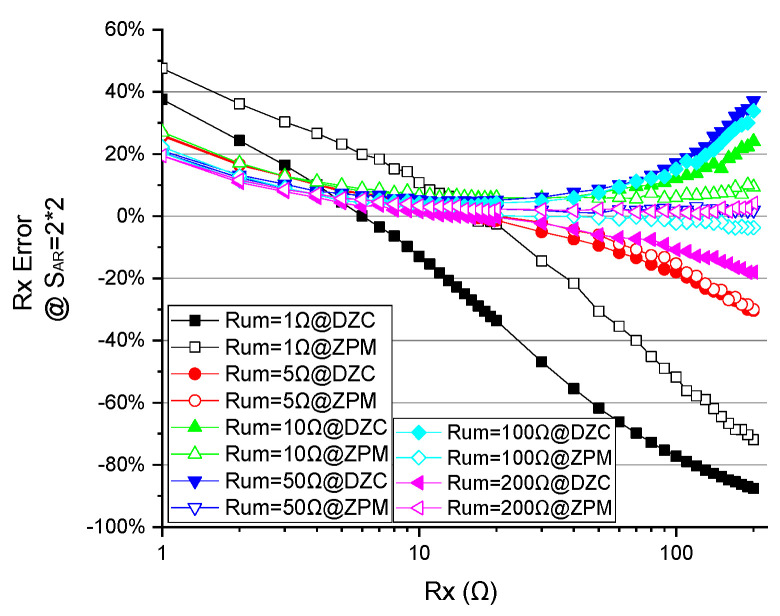
The effect of $R_{x}$ on measurement error of different $R_{um}$ when $S_{AR}=2\times 2$ in DZCM and ZPM (EXP B).

In the case of $R_{um}=1\;Ω$ (Figure 7b),

i. *e%* of the DZCM is smaller than that of the ZPM within the $R_{x}$ range of 1 Ω to 10 Ω, showing the advantage of the DZCM feedback network in bringing down the crosstalk current.
ii. As $R_{x}$ increases from 10 Ω to 200 Ω, measurement error changes from zero to a significant negative value. This unfavorable event also occurs in ZPM circuitry. Moreover, this event occurred in reference [29] Figure 4, Figure 5, Figure 6 and Figure 7 reference [39] Figure 8, reference [21] Figure 9, reference [36] Figure 5, reference [34] Figure 5, reference [30] Figure 9. We name this event the singular values effect (SVE), as it occurs when the measured resistor is tremendously different from the adjacent unmeasured resistors.

A simple 2 × 2 array (see Figure 8) is illustrated to explain the SVE. Based on Figure 8, in the extreme case of $R_{x}=R_{11}=200\;Ω$, current flowing through $R_{11}$ decreases, denoted by $I_{11}=V_{in}/R_{x}=10\;\mathrm{mV}/200\;Ω=50\;\mu A$. Meanwhile, for the adjacent resistor on the same row, $R_{12}=1\;Ω$. The current through $R_{12}$, represented by $I_{12}$, is equivalent to $V_{in}/R_{um}=10\;\mathrm{mV}/1\;Ω=10\;\mathrm{mA}$. Our measurement shows that the parasitic resistances resulting from the cable linking the sensor to PCB are $R_{par}=0.01\;Ω$ and the voltage at column 2 is $V_{Col2}=10\;\mathrm{mA}\times 0.01\;Ω=0.1\;\mathrm{mV}$, and the voltage at column 1 is $V_{Col1}\approx 0\;\mathrm{mV}$. Due to this potential difference between $V_{Col1}$ and $V_{Col2}$, there will be a crosstalk current through $R_{22}$ and $R_{21}$, $I_{22-21}=0.1\;\mathrm{mV}/2Ω=50\;\mathrm{uA}$. As $I_{22-21}$ are in the same order as $I_{11}$, the measurement error can add up to 100%.

Figure 7 also shows the improvement of the DZCM in reducing measurement error in the range of $1\;Ω<R_{x}<10\;Ω$ and $R_{um}=1\;Ω$. That is to say, in low resistance RSA, the DZCM is capable of decreasing the measurement error.

**EXP B**:

As shown in Figure 9, the *e%* of the DZCM is smaller or similar to the ZPM in the range of $1\;Ω<R_{x}<10\;Ω$. This measurement error in the DZCM becomes larger than the ZPM as $R_{x}$ increases to the range of $10\;Ω<R_{x}<200\;Ω$, the increment has been explained in EXP A above as SVE.

**Figure 10:**
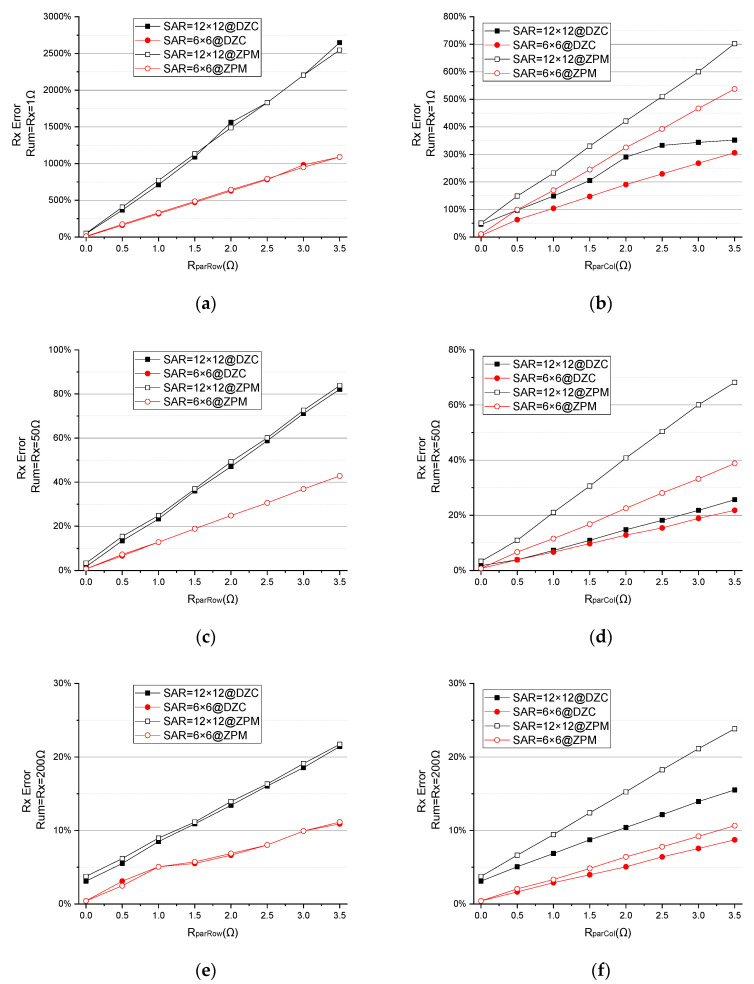
The effect of $R_{par}$ on measurement error of different $S_{AR}=6\times 6,\;12\times 12$ when $R_{x}=R_{um}=1\;Ω,\;50\;Ω,\;200\;Ω$. (**a**,**c**,**e**) Effect of $R_{parRow}$ and (**b**,**d**,**f**) effect of $R_{parCol}$ in DZCM and ZPM (EXP C).

**EXP C**:

As shown in Figure 10a,c,e, when $R_{parRow}\;$(the parasitic resistor of row wires) changes from 0 Ω to 3.5 Ω and $R_{um}=R_{x}=1\;Ω,\;50\;Ω,\;200\;Ω$, the $R_{x}$ error of the ZPM is the same as in the DZCM.

When $R_{parCol}$ (the parasitic resistor of column wires) changes from 0 Ω to 3.5 Ω, $R_{um}=R_{x}=1\;Ω,\;50\;Ω,\;200\;Ω$, as demonstrated in Figure 10b,d,f, the $R_{x}$ error of the ZPM is higher than that of the DZCM. The larger array size results in a greater error difference between the ZPM and DZCM. This error difference increases as $R_{parCol}$ goes up. This is due to the presence of the negative feedback network in the DZCM that effectively reduces the parasitic effect of $R_{par}$ in the low resistance domain.

**EXP D**:

To analyze the system performance with regard to offset voltage, error variation is preferred over absolute error. Absolute error, as discussed in EXP A, can be nulled by manual operation. On the other hand, the fluctuation of *e%* is non-zero, as $v_{osC}$ (offset voltage of column amplifiers) and $v_{osR}$ (offset voltage of row amplifiers) change. This inconstancy of measurement error often results in temperature drift and process variation of amplifier chips.

Offset voltages exist across the rows and columns in RSA. In the experiment to examine the effect of $v_{osC}$, $v_{osR}$ is kept constant at zero and $v_{osC}$ changes from −3 mV to 3 mV in steps of 1 mV. Similarly, for the second study to evaluate the influence of $v_{osR}$, $v_{osC}$ is fixed to zero and $v_{osR}$ is varied from −3 mV to 3 mV in steps of 1 mV.

The offset voltages $v_{osR}$ and $v_{osC}$ are applied to the positive inputs of all row and column amplifiers, labeled as $v_{os1}$ and $v_{os2}$, respectively (see Figure 4a,b).

Error variation is defined as the difference in measurement error associated with experimental conditions.

**Figure 11:**
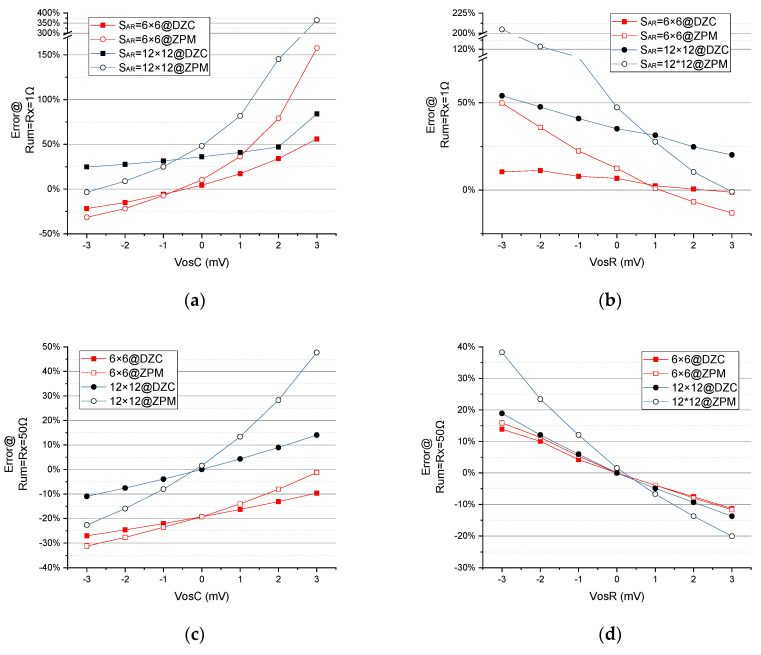

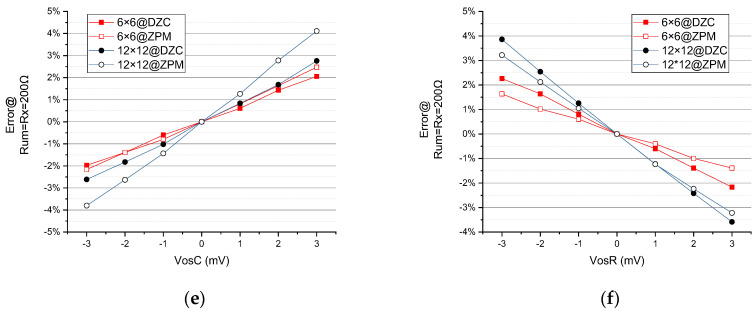
The effect of (**a**,**c**,**e**) $v_{osC}$ and (**b**,**d**,**f**) $v_{osR}$ on measurement error of different $S_{AR}=6\times 6,\;12\times 12$ when $R_{x}=R_{um}=1\;Ω,\;50\;Ω,\;200\;Ω$ (EXP D).

As seen in Figure 11a–e, when $v_{osC}$ and $v_{osR}$ change from −3 mV to +3 mV with 1 mV steps, the DZCM (the curve with solid dots) has lower $R_{x}$ error variation than the ZPM (the curves with open dots) in all array sizes. This reveals that the DZCM is capable of eliminating the adverse effects arising from $v_{osR}$ and $v_{osC}$ of row/column amplifiers. Such improvements can be proven in Equation (5) and Equation (1b), respectively. The DZCM Equation (5) has one less item than ZPM Equation (6). That is because, from Equation (5), the presence of $A_{f}$ in the DZCM feedback network helps to reduce the effect of offset voltage on the error measurement.

As seen in Figure 11f, when $v_{osR}$ changes from −3 mV to +3 mV with 1 mV steps, the DZCM has larger $R_{x}$ error variation than the ZPM in all array sizes. This reveals that the DZCM is not suitable for high value RSA. However, the error gaps between the DZCM and ZPM decrease with array size increases. This implies the DZCM will have better performance when the high value RSA has a larger array size.

Table 2 shows these performances.

### 3.2. DZCM and ZPM Application on Liquid Metal EGaIn Based Flexible RSA

**Figure 12:**
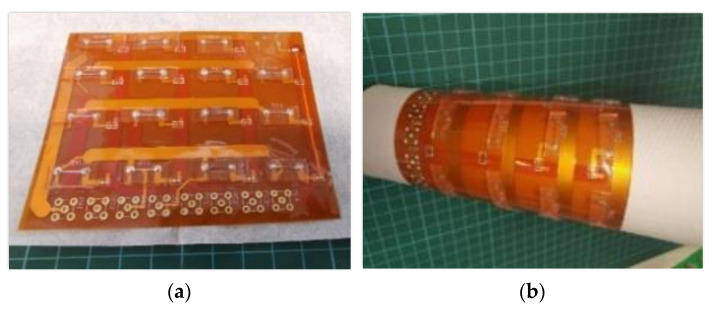
EGaIn based 4 × 4 flexible RSA (**a**) on flat surface and (**b**) on bending surface.

A liquid metal EGaIn based wearable 4 × 4 RSA was fabricated [5,7], as displayed in Figure 12.

**Figure 13:**
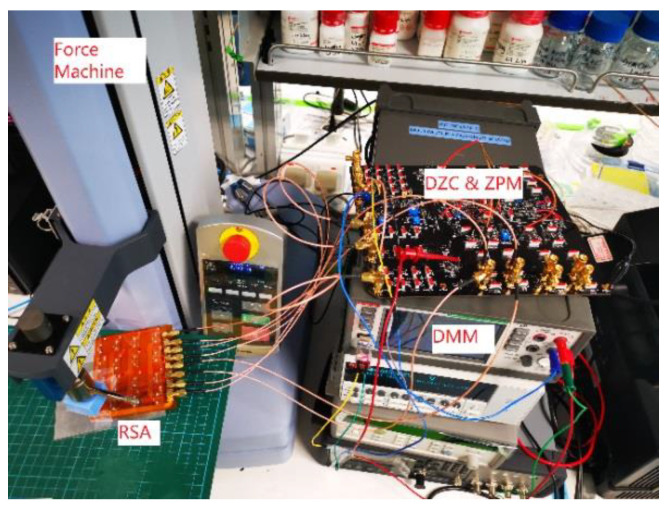
Force sensor test bench.

SHIMADZU EZ-SX was used in the mechanical testing to apply 0 N to 2 N loading onto the RSA. A KEITHLEY DMM 6500 digital multimeter was used to measure the single resistor values and the output voltage of DZCM/ZPM circuits. Figure 13 shows the experimental setup.

We firstly indented a single independent sensor and measured its resistance directly. This sensor was then connected to a number of other sensors to form a 4 × 4 flexible RSA with DZCM and ZPM readouts. Subsequently, a force was applied onto this particular sensor. Output voltages of the DZCM and ZPM were recorded. These voltage values were converted to resistance by Equation (1).

**Figure 14:**
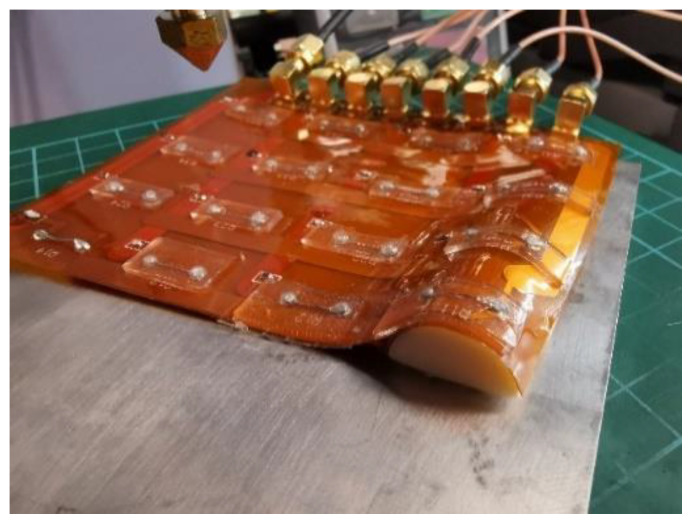
Flexible sensor mounted onto bending surface with thumb-like size.

We tested the single sensor and flexible RSA on flat and curved surfaces. The curved surface was shaped by a thumb sized semi-cylinder as shown in Figure 14.

In total, we conducted six experiments to assess the performance of the DZCM and its improvement on the actual EGaIn based RSA. The experimental layouts were:

i. Single sensor on a flat surface;
ii. single sensor on a curved surface;
iii. RSA with ZPM on a flat surface;
iv. RSA with ZPM on a curved surface;
v. RSA with DZCM on a flat surface;
vi. RSA with DZCM on a curved surface.

**Figure 15:**
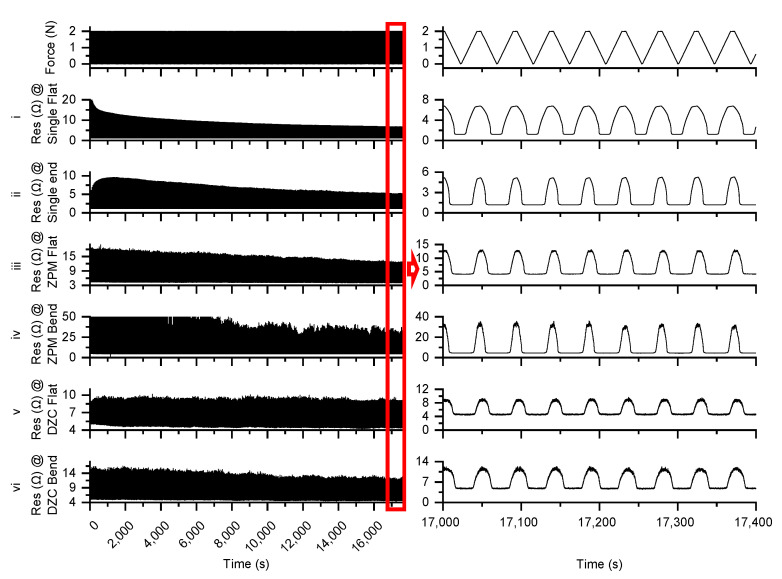
Five hours of measurement results.

We continuously indented the sensor for about 5 h. Figure 15 shows the 5 h measurement results of the above experiments.

As shown in Figure 15, experiments (i) and (ii) tested only one independent sensor. Both experiments have resistance values that decrease with time and eventually stabilize at 6 Ω on a flat surface and 5 Ω on a curved surface. This is the characteristic of EGaIn based flexible sensors [5]. Experiments (iii) and (iv) tested the RSA with a ZPM readout. The resistance values decreased with time and stabilized at 12 Ω on the flat surface and 30 Ω on the curved surface. Experiments (v) and (vi) tested the RSA with a DZCM readout. The resistance values acquired were the most stable, amounting to 9 Ω on the flat surface and 10 Ω on the curved surface.

From Figure 15, we can conclude two benefits from the DZCM design:

The DZCM has less measurement error than the ZPM on both flat and curved surfaces, especially when the array has low resistor value.

Measurement data from DZCM readout were more stable than that of the ZPM in about 4 h.

### 3.3. Discussion

It is a challenge to create low value RSAs for low power applications due to the lack of a compatible and stable readout design. Due to the low resistance value and the huge number of resistors involved in an RSA, driving voltage has to be minimized to achieve low power consumption, then the signal level is low. Thus, the noise of the parasitic resistance coming from the connection wires and the input offset voltage of the amplifiers will increase the measurement error greatly. Finally, it leads to a low signal to noise ratio (SNR).

To achieve low power consumption in the RSA, we used: ±2.5 V supply power and 10 mV driving voltage.

To decrease the parasitic wire resistance, we used:

i. Copper wires of 3.6 mm/35 mm/70 µm (width/length/thickness) to connect the resistors to form an RSA on the PCB.
ii. SMA coaxial connector/cable to link RSA PCB to the readout PCB.
iii. Copper wires of 0.15 mm/75 mm/70 µm (width/length/thickness) to connect the SMA connectors and amplifiers on the PCB in the EXP.

To decrease the input offset voltage of amplifiers, we used: OPA4388 with 0.25 µV offset voltage.

As demonstrated in Figure 10 and Figure 11, the DZCM is able to decrease measurement error arising from the parasitic resistance of wires and the input offset voltage of amplifiers. These characteristics of the DZCM, as explained below, are very important to realize steady and reliable wearable applications.

i. The larger physical dimensions of an electrical connection lead to lower parasitic resistance. Nevertheless, the use of bulky connecting wires and connectors in wearable systems is not feasible as they defeat the purpose of making an accessory, which is supposed to be comfortable and easy to wear. The DZCM helps to solve this issue as it has higher tolerance for parasitic resistance. In other words, it enables thinner wires and smaller connectors to be used in the wearable.
ii. The input offset voltage of the amplifier varies with the ambient temperature. Consequently, the measurement result is greatly influenced by the operating environment. The input offset voltage also varies in mass production; thus, the measurement result changes with different product batches. By applying the DZCM, that is less sensitive to the fluctuation of the input offset voltage, these issues can be easily resolved.

The low driving voltage required in the DZCM is another advantage which enables low power consumption. This enhances the wearable’s performance.

The DZCM is cost effective and most likely to be useful in RSA with large array size and high resistance values as well. We did not examine the proposed circuitry in high value RSAs. The crosstalk and parasitic effects have to be quantified to prove its usability. Nonetheless, simulation data in the literature [41] show this tendency.

Lastly, the proposed DZCM design is still in its early stage of development. The measurement error from crosstalk and parasitic resistance is still significant in the readout system. The singular values effect of the DZCM, such as that shown in Figure 8, also requires further improvement.

## 4. Conclusions

We have discussed the ZPM and established its simplified models to derive the respective output voltage equations. Subsequently, we introduced a new circuit design, called the DZCM, and established a simplified model to deduce its output voltage equations. We also analyzed its measurement performance with different array sizes, input offset voltages of driving amplifier, unmeasured resistance values, parasitic resistance values and resistance value of the resistor of interest. The results show that the DZCM has lower e% than the ZPM. In terms of error variation ratio from amplifier offset voltage, the DZCM has a 4%/mV (row) to 7%/mV (column) ratio, while ZPM has an almost 25%/mV (row) to 45%/mV (column) ratio which increases with array size.

In short, the DZCM is very useful for low value RSAs used in wearable applications. This new circuitry helps to reduce measurement error in the readout system and bring down the material cost for mass production.

## 5. Patents

One Patent Pending: A circuit to measure low resistor value resistive sensor array with dynamical zero current function (10202204307R).

## Figures and Tables

**Table 1:** Experiments setup for DZCM with selected combinations.

EXP A (*R_par_* = 0 Ω, *v_OS_* = 0 mV)			EXP B (*R_par_* = 0 Ω, *v_OS_* = 0 mV, *S_AR_* = 2 × 2)		EXP C (*v_OS_* = 0 mV)			EXP D (*R_par_* = 0 Ω)		
*R_x_* (Ω)	*R_um_* (Ω)	*S_AR_*	*R_x_* (Ω)	*R_um_* (Ω)	*R_x_* = *R_um_ (*Ω)	*R_parCol_* *R_parRow_* (Ω)	*S_AR_*	*R_x_* = *R_um_ (*Ω)	*S_AR_*	*v_OS_* (mV)
1 to 10, step = 1. 10 to 20, step = 1. 20 to 100, step = 10. 100 to 200, step = 10.	1 200	2 × 2 4 × 4 8 × 8	1 to 10, step = 1. 10 to 20, step = 1. 20 to 100, step = 10. 100 to 200, step = 10.	1 5 10 50 100 200	1 50 200	0, 0.5 1,1.5 2, 2.5 3, 3.5	6 × 6 12 × 12	1 50 200	6 × 6 12 × 12	0 ±1.0 ±2.0 ±3.0

**Table 2:** $R_{x}$ error variation by $v_{osR}$, $v_{osC}$ with $S_{AR}=6\times 6,\;12\times 12$.

$R_{x}=R_{um}(Ω)$	Array Size	$\mathrm{Error}\;\mathrm{Variation}\;\mathrm{Ratio}\;\mathrm{of}\;v_{osR}(\%/\mathrm{mV})$		$\mathrm{Error}\;\mathrm{Variation}\;\mathrm{Ratio}\;\mathrm{of}\;v_{osC}(\%/\mathrm{mV})$	
		DZCM	ZPM	DZCM	ZPM
**1**	**6** **×** **6**	1.83	9.82	12.16	29.55
	**12** **×** **12**	4.18	25.37	7.32	45.45
**50**	**6** **×** **6**	3.59	3.93	3.08	5.30
	**12** **×** **12**	4.66	8.34	3.57	10.05
**200**	**6** **×** **6**	0.63	0.43	0.58	0.66
	**12** **×** **12**	1.06	0.92	0.77	1.13

**Figure A1:**
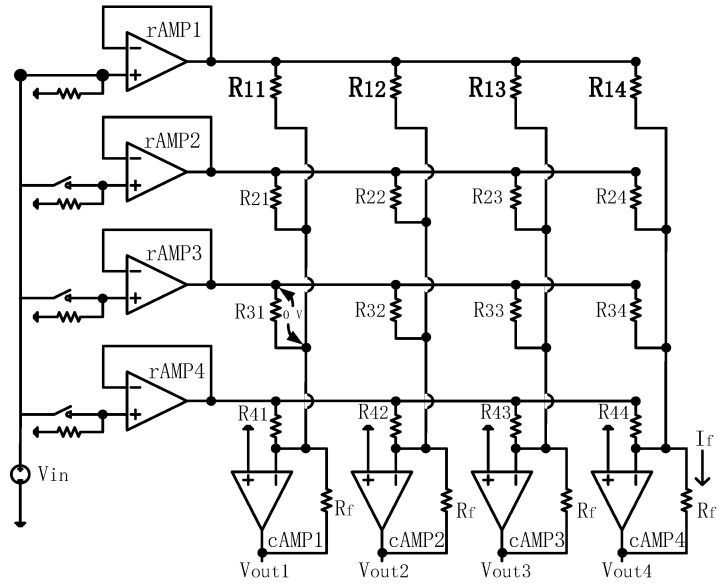
Circuit based on the ZPM.

**Figure A2:**
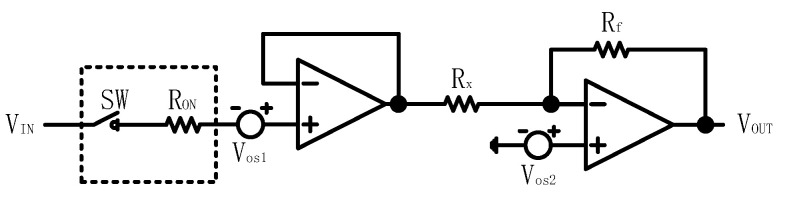
Circuit designs that include parasitic effects in the cases of ZPM.

**Figure A4:**
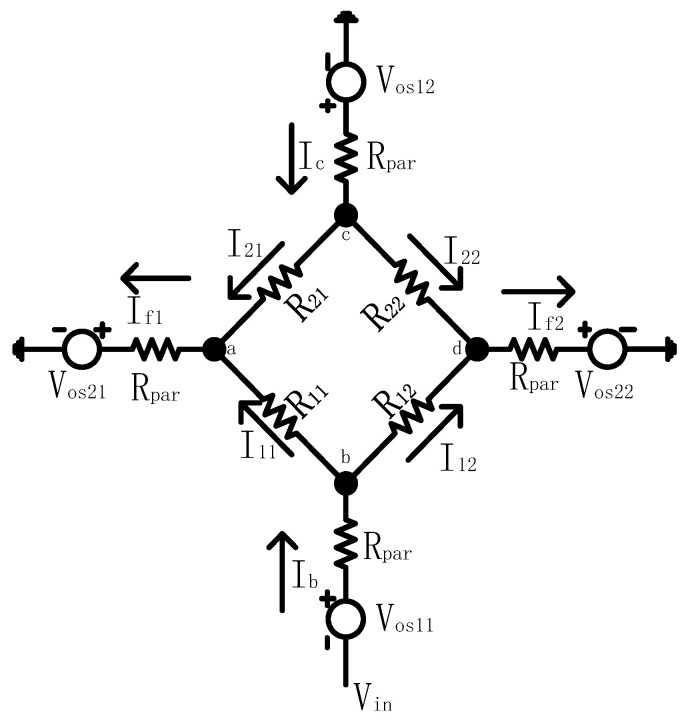
Array network extracted and analyzed with Kirchhoff’s law.

## Data Availability

Not applicable.

## Appendix A

One ZPM structure is shown in Figure A1. There are one row driving amplifier (rAMP) in each row and one column driving amplifier (cAMP) in each column. Resistor $R_{1i}$ (*i* = 1, 2, 3, 4) is connected to two amplifiers on each of its nodes. The resistor is driven to $V_{in}$ by rAMP1 and grounded by cAMPi. $V_{outi}$ is generated by cAMPi which drives one node of $R_{1i}$ to ground.

Two nodes of the other adjacent unmeasured resistors $R_{um}$ ($R_{21}$ to $R_{44}$) are grounded through their connected rAMP and cAMP. The example is $R_{31}$ in Figure A1, and these resistors have zero potential difference, thus there will be no current flow through $R_{um}$. Then, the crosstalk current flowing through $R_{um}$ will be cut off. The ideal $R_{xi}$ measurement value can be computed from Equation (A1).

(A1) $$R_{1i}=\frac{V_{in}}{I_{f}}=-\frac{V_{in}}{V_{outi}}\times R_{f}\;$$

$R_{1i}$ is the measured resistor, $V_{in}$ is the input voltage to drive the measured resistor, $I_{f}$ is the current of the feedback resistor $R_{f}$ of the column amplifier, $V_{outi}$ is the output voltage of the column amplifier.

In the case of an actual hardware system, the parasitic effect must be taken into consideration. The ON state switch resistor ($R_{ON}$) and the amplifier offset voltage ($V_{os1}$, $V_{os2}$) are included in the calculation. To include the parasitic effect and explain the characteristics of the ZPM, we simplify the network to just one resistor $R_{x}$. The revised circuit is shown in Figure A2.

The measurable resistance in Equation (A1) is modified and the new equations are depicted in Equation (A2):

(A2) $$R_{x}=-\left(\frac{V_{in}+V_{OS1}-V_{OS2}}{V_{out}}\times R_{f}\right)$$

Figure A3 shows an example of a 2 × 2 RSA, taking into account both the crosstalk and parasitic effect. In order to include non-ideal factors (row and column parasitic resistance $R_{par}\;$and amplifier offset voltage $V_{OS}$) in this model, we use the ZPM for the analysis below. Non-ideal factors are illustrated as $R_{par}\;$and $V_{OS}$ in the circuit model.

To calculate the intrinsic crosstalk current effect, we first extract the array network and apply Kirchhoff’s law, as shown in Figure A4 and Equation (A3).

(A3a) $$V_{a}=V_{os21}+I_{f1}\cdot R_{par}=V_{os21}+\left(I_{21}+I_{11}\right)\cdot R_{par}\;$$

(A3b)
$$V_{b}=V_{os11}+V_{in}-I_{b}\cdot R_{par}=V_{os11}+V_{in}-\left(I_{12}+I_{11}\right)\cdot R_{par}$$

(A3c)
$$V_{c}=V_{os12}-I_{c}\cdot R_{par}=V_{os12}-\left(I_{21}+I_{22}\right)\cdot R_{par}$$

(A3d)
$$V_{d}=V_{os22}+I_{f2}\cdot R_{par}=V_{os22}+\left(I_{12}+I_{22}\right)\cdot R_{par}\;$$

(A3e)
$$V_{b}-V_{a}=I_{11}\cdot R_{11}$$

(A3f)
$$V_{c}-V_{a}=I_{21}\cdot R_{21}\;$$

(A3g)
$$V_{b}-V_{d}=I_{12}\cdot R_{12}$$

(A3h)
$$V_{c}-V_{d}=I_{22}\cdot R_{22}\;$$

We hypothesize $R_{11}=R_{x}$ and $R_{12}=R_{21}=R_{22}=R_{um}$ and $10\cdot R_{par}<R\approx R_{x}$. After substituting (A3a, A3b, A3c, A3d) with (A3e, A3f, A3g, A3h), we obtain Equation (A4).

(A4a) $$I_{11}\cdot \left(R_{x}+2R_{par}\right)+I_{12}R_{par}+I_{21}R_{par}=V_{os11}-V_{os21}+V_{in}$$

(A4b)
$$I_{11}R_{par}+I_{21}\cdot \left(R_{um}+2R_{par}\right)+I_{22}R_{par}=V_{os12}-V_{os21}$$

(A4c)
$$I_{11}R_{par}+I_{12}\cdot R_{um}-I_{22}R_{par}=V_{os11}-V_{os22}+V_{in}$$

(A4d)
$$-I_{12}R_{par}+I_{21}R_{par}+I_{22}\cdot R_{um}=V_{os12}-V_{os22}$$

Equation (A4) is a non-homogeneous linear equation R·I=V and can be written as Equation (A5).

(A5)
$$\left(\left.\begin{array}{ccc} R_{x}+2R_{par} & R_{par} & \begin{array}{cc} R_{par} & 0 \end{array}\\ R_{par} & 0 & \begin{array}{cc} R_{um}+2R_{par} & R_{par} \end{array}\\ \begin{array}{c} R_{par}\\ 0 \end{array} & \begin{array}{c} R_{um}+2R_{par}\\ -R_{par} \end{array} & \begin{array}{cc} \begin{array}{c} 0\\ R_{par} \end{array} & \begin{array}{c} -R_{par}\\ R_{um}+2R_{par} \end{array} \end{array} \end{array}\right|\begin{array}{c} V_{os11}-V_{os21}+V_{in}\\ V_{os12}-V_{os21}\\ \begin{array}{c} V_{os11}-V_{os22}+V_{in}\\ V_{os12}-V_{os22} \end{array} \end{array}\right)$$

We define $R_{um}+2R_{par}=R_{u2}^{\text{’}}$ and$\;R_{x}+2R_{par}=R_{x2}^{\text{’}}$, then we have:

(A6)
$$\left(\left.\begin{array}{cc} \begin{array}{c} R_{x2+}\\ R_{par}\\ R_{par} \end{array} & \begin{array}{c} R_{par}\\ 0\\ R_{u2+} \end{array}\\ 0 & -R_{par} \end{array}\begin{array}{c} R_{par}\\ R_{u2+}\\ \begin{array}{c} 0\\ R_{par} \end{array} \end{array}\begin{array}{c} 0\\ R_{par}\\ \begin{array}{c} {-R}_{par}\\ R_{u2+} \end{array} \end{array}\right|\begin{array}{c} V_{os11}-V_{os21}+V_{in}\\ V_{os12}-V_{os21}\\ \begin{array}{c} V_{os11}-V_{os22}+V_{in}\\ V_{os12}-V_{os22} \end{array} \end{array}\right)$$

After rearranging the linear equation, we have:

(A7)
$$\left(\left.\begin{array}{ccc} 1 & 0 & \begin{array}{cc} 0 & 0 \end{array}\\ 0 & 1 & \begin{array}{cc} 0 & 0 \end{array}\\ \begin{array}{c} 0\\ 0 \end{array} & \begin{array}{c} 0\\ 0 \end{array} & \begin{array}{c} \begin{array}{cc} 1 & 0 \end{array}\\ \begin{array}{cc} 0 & 1 \end{array} \end{array} \end{array}\right|\begin{array}{c} \frac{\left[\left(V_{os12}-V_{os21}\right)\left(R_{um}R_{x}-R_{u2}^{\prime }R_{x2}^{\prime }\right)+R_{u2}^{\prime }\left(V_{os11}-V_{os21}+V_{in}\right)R_{par}\right]}{R_{par}R_{um}R_{x}}\\ \frac{\left[\left(V_{os12}-V_{os22}\right)\left(R_{um}^{2}R_{x}-R_{x}R_{u2}^{\prime 2}\right)+R_{x}R_{par}\left(V_{os11}+V_{os21}+V_{in}\right)R_{u2}^{\prime }\right]}{R_{par}R_{um}^{2}R_{x}}\\ \begin{array}{c} -\left[\left(V_{os11}-V_{os22}+V_{in}\right)R_{par}-\left(V_{os12}-V_{os21}\right)\left(R_{x2}^{\prime }\right)\right]/R_{um}R_{x}\\ -\left[\left(V_{os11}+V_{os21}+V_{in}\right)R_{par}+\left(V_{os12}-V_{os21}\right)\left(R_{u2}^{\prime }\right)\right]/R_{um}^{2} \end{array} \end{array}\right)$$

From (A3-1), we have $I_{f1}=I_{21}+I_{11}$. Deriving from (A7) row 1 ($I_{11}$) and row 3 ($I_{21}$), and after simplifying, we have:

(A8) $$I_{f1}=\frac{\left(R_{u2}^{’}\right)\left(V_{in}+V_{os11}\right)-V_{os12}\left(R_{u2}^{’}+R_{um}+R_{x}\right)-V_{os21}\left(R_{um}+R_{x}\right)+V_{os22}R_{par}}{R_{um}R_{x}}$$

We can now evaluate $I_{f1}$ from Equation (A8) by assuming $R_{par}$, $V_{os11}$, $V_{os12}$, $V_{os21}$ and $V_{os22}$ are all equal to zero (ideal case). Equation (A8) is then modified as $I_{f1}=\frac{V_{in}}{R_{x}}$ and it matches with Equation (A1).

## Appendix B

Equation (3) is a non-homogeneous linear equation R·I=V and can be written as Equation (A9a).

(A9a) $$\left(\begin{array}{cc} R_{x}+2R_{par} & R_{par}\\ R_{par} & R_{um}+2R_{par} \end{array}|\begin{array}{c} V_{os11}+V_{in}-V_{os21}\\ V_{os11}+V_{in}-V_{os22} \end{array}\right)$$

After defining $R_{um}+2R_{par}=R_{u2}^{’}$ and $R_{x}+2R_{par}=R_{x2}^{’}$ and $V_{os11}+V_{in}=V_{i1}$, we have:

(A9b) $$\left(\begin{array}{cc} R_{x2}^{’} & R_{par}\\ R_{par} & R_{u2}^{’} \end{array}|\begin{array}{c} V_{i1}-V_{os21}\\ V_{i1}-V_{os22} \end{array}\right)$$

Rearranging row 1, we have:

(A9c) $$\left(\begin{array}{cc} 0 & 1\\ R_{par} & R_{u2+} \end{array}|\begin{array}{c} \frac{\left(V_{i1}-V_{os22}\right)R_{x2+}+V_{os21}R_{par}}{R_{x2+}R_{u2+}-R_{par}{}^{2}}\\ V_{i1}-V_{os22} \end{array}\right)$$

Rearranging row 2, we have:

(A9d) $$\left(\begin{array}{cc} 1 & 0\\ 0 & 1 \end{array}|\begin{array}{c} -\frac{\left(V_{i1}-V_{os22}\right)R_{par}+\left(V_{os21}-V_{i1}\right)R_{u2}^{’}}{R_{u2}^{’}R_{x2}^{’}-R_{par}{}^{2}}\\ \frac{\left(V_{i1}-V_{os22}\right)R_{x2}^{’}+V_{os21}R_{par}}{R_{u2}^{’}R_{x2}^{’}-R_{par}{}^{2}} \end{array}\right)$$

From Equation (2a), we have $I_{f1}=I_{11}$. From Equation (A9d) row 1 ($I_{11}$), we have:

(A9e) $$I_{f1}=I_{11}=-\frac{\left(V_{i1}-V_{os22}\right)R_{par}+\left(V_{os21}-V_{i1}\right)R_{u2}^{’}}{R_{x2}^{’}R_{u2}^{’}-R_{par}{}^{2}}$$
